# Evaluation of the Association of *COMT* Rs4680 Polymorphism with Swimmers’ Competitive Performance

**DOI:** 10.3390/genes12101641

**Published:** 2021-10-19

**Authors:** Piotr Zmijewski, Agata Leońska-Duniec, Aleksander Stuła, Marek Sawczuk

**Affiliations:** 1 Faculty of Physical Education, Jozef Pilsudski University of Physical Education in Warsaw, 00-809 Warsaw, Poland; 2 Faculty of Physical Education, Gdansk University of Physical Education and Sport, 80-336 Gdansk, Poland; agata.leonska-duniec@awf.gda.pl; 3 Department of Physical Education and Physiotherapy, Opole University of Technology, 45-758 Opole, Poland; a.stula@poczta.onet.pl; 4 Institute of Physical Culture Sciences, University of Szczecin, 70-453 Szczecin, Poland; marek.sawczuk@usz.edu.pl

**Keywords:** swimming, sport genetics, polymorphism, dopamine, cognitive abilities

## Abstract

Swimmers’ competitive performance is a result of complicated interactions between physiological, biochemical, physical and psychological factors, all of which are strongly affected by water. Recently, great attention has been paid to the role of genetic factors such as the catechol-O-methyltransferase gene (*COMT*) influencing motivation, emotions, stress tolerance, self-control, sleep regulation, pain processing and perception, addictive behaviour and neurodegeneration, which may underlie differences in achieving remarkable results in sports competition. Thus, this study was performed to investigate the association between the *COMT* Val158Met (rs4680) polymorphism and athletic performance in Caucasian swimmers. A total of 225 swimmers (171 short distance (SDS) and 54 long distance swimmers (LDS)) of national or international competitive standard and 379 unrelated sedentary controls were genotyped using real-time polymerase chain reaction (real-time PCR). We found no significant differences in genotypic or allelic distributions between (1) male and female athletes; (2) SDS and LDS; (3) all athletes and sedentary controls (under codominant, dominant, recessive, and overdominant genetic models). No association was found between the *COMT* rs4680 polymorphism and elite swimming athlete status of the studied population. However, more replication studies are needed.

## 1. Introduction

An individual’s physical performance is dependent on a complex combination of developmental, behavioural and environmental factors, in addition to which genetic background plays a fundamental role in determining athletic ability [1]. The reported heritability of athlete status was estimated at around 66% [2]. The group of genetic factors that are likely to play an important role in physical performance includes numerous gene variants that influence power, strength, endurance, flexibility, muscle tissue properties, coordination, body composition and other traits [1,3,4,5]. However, a commonly neglected aspect in studies related to development of athletic ability is the association between genetic polymorphisms and cognitive abilities such as motivation, stress tolerance and self-control [6].

A principal factor involved in the regulation of cognitive abilities is the midbrain dopaminergic system. Dopamine (DA; 3,4-dihydroxy-phenylethylamine) is a catecholamine neurotransmitter involved in the development of fatigue, which leads to a decrease in intensity or interruption of exercise, through the modulation of circuits linked to the motor control and thermoregulatory as well as motivation and reward system [7,8,9]. First in rats and later in humans, an association between DA system genes and physical activity-related behaviours was confirmed [6,8,9,10]. In a study examining the association of three DA system genes with swimmers’ competitive performance, Abe et al. [6] obtained statistically significant results only for the Val158Met polymorphism within catechol-O-methyltransferase gene (*COMT*). Thus, they suggested that it is the most likely candidate gene associated with individual differences in cognitive abilities which may underlie differences in achieving remarkable results in professional sports competition and everyday physical activity [6]. However, little is known about its role in the development of physical performance in different populations, and more replication studies are required.

The human *COMT* gene located on the long arm of chromosome 22 (*locus* 22q11.21) encodes catechol-O-methyltransferase (COMT; EC 2.1.1.6), which is the main regulator of dopaminergic and adrenergic neurotransmission. In the presence of magnesium (Mg^2+^) the COMT enzyme transfers a methyl group from S-adenosyl-L-methionine (SAM) to one of the catecholic hydroxyls. The O-methylation activity has a role in the inactivation of catecholamine neurotransmitters and catechol hormones such as DA [11]. Two isoforms expressed from different promoters are distinguished: the soluble form (S-COMT) and the membrane-bound form (MB-COMT). The second isoform is expressed mainly in brain neurons and is involved in the regulation of extracellular DA levels in the prefrontal cortex. Although its role was established in 1958 [12], the COMT enzyme’s function in other pathways and diseases has become an increasingly popular subject of study in recent years. It has been demonstrated to be involved in neuropsychiatric disorders and neurobiology of cognition, emotions, behaviour, sleep regulation, pain processing and perception, addictive behaviour and neurodegeneration [11,13] (Table 1). Therefore, the enzyme’s activity may also play a key role in the development of sport abilities.

Regarding its important physiological role in catecholamine catabolism, the polymorphisms affecting the enzyme expression have been widely studied. The main cause of variation in the enzyme’s activity is a common single nucleotide polymorphism (SNP) within the coding region of the *COMT* gene involving a G to A transition, resulting in the substitution of the 158th amino acid residue of MB-COMT from valine to methionine (Val158Met; rs4680). The presence of the Met allele has been shown to decrease the activity of the COMT enzyme, due to its higher thermolability, which in turn increases the DA levels [15]. This SNP has been positively associated with swimmers’ competitive performance in an Asian population [6]; the current study was aimed to verify if the association exists in Polish (Caucasian) population and extend the research in the swimming athletic group of different training goals (long-versus short-distance performance). However, more replication studies in different populations are needed.

Considering the key role of the DA system and its inhibitors in the regulation of cognitive abilities which may underlie differences in the potential to be an elite athlete, the aim of the present study was to examine the genotype and alleles frequencies of the *COMT* rs4680 polymorphic site in elite short- (SDS) and long-distance swimmers (LDS), as well as sedentary controls.

## 2. Materials and Methods

### 2.1. Participants

The study group consisted of 225 professional swimmers (106 females and 119 males; 20.3 ± 2.7 years) of Caucasian origin. The recruited athletes competed in national and international competition and achieved ≥600 FINA points, which differentiates athletes from amateurs. The mean swimming performance considered as the personal best result was 734 points. All participants were finalists of the Polish National Championships; 7 of them had taken part in the Olympic Games and 48 in the European Championships or World Championships. The study group included:

- World Championship medallists (*n* = 8),
- European Championship medallists (*n* = 15),
- Polish Championship medallists (*n* = 202).

The recruited swimmers were divided into two subgroups, according to their competitive distance:

- Short distance swimmers (SDS; competitive distance 50–200 m; *n* = 169,
- Long distance swimmers (LDS; competitive distance ≥400 m; *n* = 56.

The control group consisted of 379 (157 females and 222 males; 22.6 ± 2.8 years) unrelated Caucasian college students with no background in professional sport.

The experimental protocols were approved by the Pomeranian Medical University Ethics Committee in Poland. All procedures were conducted ethically according to the Strengthening the Reporting of Genetic Association studies statement (STREGA) and the World Medical Association Declaration of Helsinki.

### 2.2. Genetic Analyses

Total DNA was isolated from the buccal cells by a GenElute Mammalian Genomic DNA Miniprep Kit (Sigma, Steinheim, Germany) according to the producer’s protocol. All samples were genotyped in duplicate. An allelic discrimination assay on a C1000 Touch Thermal Cycler (Bio-Rad, Feldkirchen, Germany) instrument with TaqMan probes was applied. To discriminate the *COMT* rs4680 alleles, we used TaqMan Pre-Designed SNP Genotyping Assays (Applied Biosystems, Waltham, MA, USA) (assay ID: C__25746809_50), consisting of fluorescently labelled (FAM and VIC) minor groove binder (MGB) probes and two specific primers.

### 2.3. Statistical Analyses

Allele frequencies were calculated using observed genotype counts. The association of the COMT polymorphism was analysed using a generalised linear model framework under codominant, dominant, recessive and overdominant models. The analysis included sex and swimming distances (SDS, LDS). Genetic models were defined with respect to the minor allele. Data analysis was done using R software (version 4.0.3, https://r-project.org, accessed on 13 August 2021), and *p* values < 0.05 were considered significant.

## 3. Results

The *COMT* rs4680 genotype frequencies were in concordance with the Hardy–Weinberg equilibrium in both athletes (*p* = 0.226) and control individuals (*p* = 0.149). There were no significant differences in genotypic or allelic distribution for both sex and competitive distance, so we decided to pool the sample, as this allowed the statistical power to be increased. Additionally, no association of the rs4680 *COMT* polymorphism with athletic status was found regardless of the underlying genetic model (Table 2).

## 4. Discussion

Swimmers’ competitive performance is a result of complicated interactions between physiological, biochemical, biomechanical, physical and psychological factors, all of which are strongly affected by water [16]. Although swimming research has underlined energetics and biomechanical assessment to understand the specific needs of this discipline, recently, special interest has been given to sports genomics focusing on the organization and functioning of the elite athletes’ genome [3,16]. Despite a rather high heritability of athlete status [2], the search for genetic markers affecting elite sport predispositions remains a challenging task [3,17]. In the last years, great attention has been paid to the role of genetic factors influencing cognitive abilities such as motivation, stress tolerance and self-control, which may underlie differences in achieving remarkable results in sports competition. One candidate genetic marker of the dopaminergic system, with a confirmed association with physical activity-related behaviours, is *COMT*, yet little is known about its role in physical performance development [6].

In this study, we assessed the genotype distributions and allele frequencies of the *COMT* rs4680 polymorphism between swimmers of Caucasian origin divided into groups based on their competitive distance (SDS and LDS), as well as a control group. Unfortunately, we did not find significant differences in the genotype distributions or allele frequencies between (1) male and female athletes; (2) SDS and LDS; (3) all athletes and sedentary controls. Thus, the main finding of this study is the lack of an association between the *COMT* rs4680 polymorphism and elite athlete status. However, it should be noted that more replication studies are needed to establish the role of the polymorphism in the development of elite swimming performance.

Previously, in a study including 57 Asian male elite athletes, Abe et al. [6] examined the interaction between the genetic polymorphisms influencing DA system functions and swimmers’ competitive performance. They observed a significant effect of the *COMT* rs4680 polymorphism on FINA points, which indicates a swimmer’s competitive performance. The carriers of the Met allele showed a higher mean value of FINA points and were more often elite athletes than the carriers of the Val allele. They suggested that swimmers with the Met allele might achieve outstanding sports results under high pressure due to their superior executive control. However, no effect of the *DRD2* rs1800497 and *DRD3* rs6280 polymorphisms on FINA points was observed [6]. Another study performed on 75 participants undergoing 17 weeks of running training demonstrated that individuals with the Val/Val genotype showed superior executive control abilities after aerobic exercise training compared to the Met allele carriers. It was concluded that a rise in physical fitness causes improved cognitive functioning via dopaminergic modulation [14]. Abe et al. [6] suggested that improving the athletic performance of Val/Val swimmers might be possible by applying an intensive training program to improve aerobic capacity. However, our results did not confirm significant differences in the genotype distributions or allele frequencies between elite swimmers and sedentary controls, or SDS and LDS.

Recent studies have also demonstrated that COMT is estrogenically catabolised, and a significant sex-by-genotype interaction on psychiatric phenotype, cognitive abilities and personality traits has been described [18,19]. The expression of COMT in the prefrontal cortex has been shown to be higher in males than females, and is linked to enhanced susceptibility to the Val/Val genotype effects in men [11,18]. The obtained results also did not the confirm sexually dimorphic effects of *COMT* in the group of Caucasian swimmers.

Unfortunately, the present study has some potential limitations, which could have influenced the results. One of them is the lack of a direct evaluation of executive control abilities with objective tests. Secondly, information about individuals’ FINA points was unavailable, so further studies are needed. Third, the statistical analysis of the relatively small group of Polish elite swimmers might not have sufficient statistical power to yield reliable results. Finally, we examined only one candidate genetic marker, but in the future, we plan to extend the analysis to new genes of the DA system which, individually or in haplotype combination, may influence physical activity-related behaviours.

The relatively high frequency of the *COMT* rs4680 polymorphism, together with its key role in regulating catecholamine catabolism, rapidly led to several efforts to establish its possible significance to a range of neuropsychiatric phenotypes [20]. The amino acid change (Met → Val substitution at codon 158) makes the enzyme susceptible to distortion of the active site and aggregation of polypeptide at physiological temperature, leading to a lower enzyme activity in Met allele carriers, whereas higher activity is observed in Val allele carriers [20,21]. The Met/Met genotype gives a three- to four-fold decrease in COMT activity relative to the Val/Val genotype, with heterozygotes demonstrating medium activity [22]. The frequency of the Met allele in diverse ethnic groups has ranged from 0.01 to 0.62, e.g., 0.49–0.54 in Caucasians, 0.49 in Southwest Asians, 0.18–0.3 in East Asians, and 0.03–0.04 in African Americans and Africans [11]. Bilder et al. hypothesised that the Met allele leads to higher tonic DA levels and reciprocal decreases in phasic DA in subcortical regions and enhanced D1 receptor transmission cortically. This mechanism may give higher stability but lower flexibility to the neural network activation states that underlie significant aspects of working memory and executive functions. These effects may be advantageous or disadvantageous depending on the phenotype, as well as numerous endogenous and environmental factors [20]. In the other study, the authors suggested that the chronic levels of physical activity, as seen in ultra-endurance athletes, show increased novelty-seeking in Met (158) homozygous allele carriers supporting the hypothesis that there is an association between personality traits and *COMT* Val158Met (rs4680) genotype [23]. The association between genetic variation and temperamental traits has been also confirmed in a study conducted by Leznicka and co-workers, in which associations between a functional polymorphism of the *COMT* gene and FCB-TI scores for temperament traits were found [24]. In this group of combat athletes, homozygous subjects with the Val allele (Val/Val) were characterised by lower sensitivity compared with the Met allele carriers (Met/Met or Met/Val) [24].

The animal experiments concerning the effect of physical activity on brain function demonstrated that exercise initiation leads to increased levels of serum calcium, which is transported to the brain, where it stimulates DA synthesis through a calmodulin-dependent system. Consequently, the higher DA levels regulate various brain functions [25]. In contrast, the decrease in DA concentration which is observed as physical activity continues probably due to the inhibitory effects of serotonin. The activity of the DA system is correlated with the development of fatigue through associated modulation circuits that are linked to thermoregulatory regulation, motor control, motivation and the reward system [7,26]. During exercise, a rise in DA system activity seems to impact tolerance to higher core temperatures before stopping exercise. This observation was confirmed by the administration of blockers of the D1 and D2 receptors, which resulted in impaired running performance by decreasing the tolerance to heat storage. The blockade also weakens the dissipation of exercise-induced heat and recovery of metabolic rate during the post-exercise period. Thus the authors suggested that the DA system and its inhibitors are essential for heat balance and exercise performance [7,27]. However, in humans, the biological mechanisms underlying the improvement of cognitive abilities correlated with exercise-induced changes in the brain are still unclear and have not been comprehensively studied, though the DA system seems to play a key role [14].

## 5. Conclusions

The study found no evidence for an association between the *COMT* rs4680 polymorphism and elite swimming athlete status. We did not observe significant differences in the genotype distributions or allele frequencies between (1) male and female athletes; (2) SDS and LDS; (3) all athletes and sedentary controls. However, more replication studies are needed.

## Figures and Tables

**Table 1:** General characteristics of the possible effect of *COMT* Val158Met (rs4680) on selected traits and sports performance.

Effect on	*COMT* Val158Met (rs4680)	
	A Allele	G Allele
Amino acid residue	Met	Val
Properties of enzyme	Higher thermolability	Normal thermolability
Enzyme activity	Low	High
Dopamine levels	High	Low
Cognitive abilities	Benefits on tasks demanding stability (maintenance phases of working memory, sustained execution of prepotent response sets), but excessive cognitive rigidity (difficulty updating or switching)	Benefits on tasks demanding flexibility (updating contents of working memory, switching to novel task), but lack cognitive stability (increased distractibility, loss of cognitive sets)
Sport	Higher mean value of FINA points and greater chances of becoming an elite athlete [6]	Superior executive control abilities after aerobic exercise training [14]

**Table 2:** Association analysis (genotypic and allelic) of the rs4689 *COMT* polymorphism with athletic status.

Genotype	Controls (*n* = 377)	Athletes (*n* = 225)	OR (95% CI)	*p*
	Codominant			
AA *	106 (28.1)	71 (31.6)	1	0.614
AG *	174 (46.2)	102 (45.3)	0.88 (0.59–1.29)	
GG *	97 (25.7)	52 (23.1)	0.80 (0.50–1.26)	
	Dominant			
AA	106 (28.1)	71 (31.6)	1	0.371
AG-GG	271 (71.9)	154 (68.4)	0.85 (0.59–1.22)	
	Recessive			
AA-AG	280 (74.3)	173 (76.9)	1	0.472
GG	97 (25.7)	52 (23.1)	0.87 (0.59–1.27)	
	Overdominant			
AA-GG	203 (53.8)	123 (54.7)	1	0.845
AG	174 (46.2)	102 (45.3)	0.97 (0.69–1.35)	
A	386 (51.2)	244 (54.2)	1	0.311
G	368 (48.8)	206 (45.8)	0.89 (0.70–1.12)	

* AA genotype corresponds to the Met/Met genotype; the GG genotype corresponds to the Val/Val genotype; the AG genotype corresponds to the Met/Val genotype.

## Data Availability

The data presented in this study are available on request from the corresponding author. The data are not publicly available due to privacy/ethical restrictions.

## References

[1] Ahmetov I.I., Fedotovskaya O.N. (2012). Sports Genomics: Current State of Knowledge and Future Directions. Cell. Mol. Exerc. Physiol..

[2] De Moor M.H.M., Spector T.D., Cherkas L.F., Falchi M., Hottenga J.J., Boomsma D.I., De Geus E.J.C. (2007). Genome-Wide Linkage Scan for Athlete Status in 700 British Female DZ Twin Pairs. Twin Res. Hum. Genet..

[3] Boulygina E.A., Borisov O.V., Valeeva E.V., Semenova E.A., Kostryukova E.S., Kulemin N.A., Larin A.K., Nabiullina R.M., Mavliev F.A., Akhatov A.M. (2020). Whole Genome Sequencing of Elite Athletes. Biol. Sport.

[4] Zmijewski P., Leońska-Duniec A. (2021). Association between the FTO A/T Polymorphism and Elite Athlete Status in Caucasian Swimmers. Genes.

[5] Leońska-Duniec A., Ahmetov I.I., Zmijewski P. (2016). Genetic Variants Influencing Effectiveness of Exercise Training Programmes in Obesity—An Overview of Human Studies. Biol. Sport.

[6] Abe D., Doi H., Asai T., Kimura M., Wada T., Takahashi Y., Matsumoto T., Shinohara K. (2018). Association between COMT Val158Met Polymorphism and Competition Results of Competitive Swimmers. J. Sports Sci..

[7] Cordeiro L.M.S., Rabelo P.C.R., Moraes M.M., Teixeira-Coelho F., Coimbra C.C., Wanner S.P., Soares D.D. (2017). Physical Exercise-Induced Fatigue: The Role of Serotonergic and Dopaminergic Systems. Braz. J. Med Biol. Res..

[8] Heyes M.P., Garnett E.S., Coates G. (1985). Central Dopaminergic Activity Influences Rats Ability to Exercise. Life Sci..

[9] Gerald M.C. (1978). Effects of (+)-Amphetamine on the Treadmill Endurance Performance of Rats. Neuropharmacology.

[10] Lee C.G., Moon H., Park S. (2020). The Effects of Dopamine Receptor Genes on the Trajectories of Sport Participation from Adolescence through Young Adulthood. Ann. Hum. Biol..

[11] Bastos P., Gomes T., Ribeiro L. (2017). Catechol-O-Methyltransferase (COMT): An Update on Its Role in Cancer, Neurological and Cardiovascular Diseases. Reviews of Physiology, Biochemistry and Pharmacology.

[12] Axelrod J., Tomchick R. (1958). Enzymatic O-Methylation of Epinephrine and Other Catechols. J. Biol. Chem..

[13] Machoy-Mokrzyńska A., Starzyńska-Sadura Z., Dziedziejko V., Safranow K., Kurzawski M., Leźnicka K., Sulżyc-Bielicka V., Jurewicz A., Bohatyrewicz A., Białecka M. (2019). Association of COMT Gene Variability with Pain Intensity in Patients after Total Hip Replacement. Scand. J. Clin. Lab. Investig..

[14] Stroth S., Reinhardt R.K., Thöne J., Hille K., Schneider M., Härtel S., Weidemann W., Bös K., Spitzer M. (2010). Impact of Aerobic Exercise Training on Cognitive Functions and Affect Associated to the COMT Polymorphism in Young Adults. Neurobiol. Learn. Mem..

[15] Qayyum A.C., Zai C., Hirata Y.K., Tiwari A., Cheema S., Nowrouzi B., Beitchman J., Kennedy L. (2015). The Role of the Catechol-o-Methyltransferase (COMT) GeneVal158Met in Aggressive Behavior, a Review of Genetic Studies. Curr. Neuropharmacol..

[16] Ben-Zaken S., Eliakim A., Nemet D., Kaufman L., Meckel Y. (2022). Genetic Characteristics of Competitive Swimmers: A Review. Biol. Sport.

[17] Youn B.-Y., Ko S.-G., Young Kim J. (2021). Genetic Basis of Elite Combat Sports Athletes: A Systematic Review. Biol. Sport.

[18] White T.P., Loth E., Rubia K., Krabbendam L., Whelan R., Banaschewski T., Barker G.J., Bokde A.L.W., Büchel C., Conrod P. (2014). Sex Differences in COMT Polymorphism Effects on Prefrontal Inhibitory Control in Adolescence. Neuropsychopharmacology.

[19] De Castro-Catala M., Barrantes-Vidal N., Sheinbaum T., Moreno-Fortuny A., Kwapil T.R., Rosa A. (2015). COMT-by-Sex Interaction Effect on Psychosis Proneness. Biomed Res. Int..

[20] Bilder R.M., Volavka J., Lachman H.M., Grace A.A. (2004). The Catechol-O-Methyltransferase Polymorphism: Relations to the Tonic-Phasic Dopamine Hypothesis and Neuropsychiatric Phenotypes. Neuropsychopharmacology.

[21] Rutherford K., Alphandéry E., McMillan A., Daggett V., Parson W.W. (2008). The V108M Mutation Decreases the Structural Stability of Catechol O-Methyltransferase. Biochim. Biophys. Acta-Proteins Proteom..

[22] Weinshilboum R.M., Otterness D.M., Szumlanski C.L. (1999). Methylation Pharmacogenetics: Catechol O-Methyltransferase, Thiopurine Methyltransferase, and Histamine N-Methyltransferase. Annu. Rev. Pharmacol. Toxicol..

[23] van Breda K., Collins M., Stein D.J., Rauch L. (2015). The COMT val158met polymorphism in ultra-endurance athletes. Physiol. Behav..

[24] Leźnicka K., Niewczas M., Kurzawski M., Cięszczyk P., Safranow K., Ligocka M., Białecka M. (2018). The association between COMT rs4680 and OPRM1 rs1799971 polymorphisms and temperamental traits in combat athletes. Pers. Individ. Differ..

[25] Sutoo D., Akiyama K. (2003). Regulation of Brain Function by Exercise. Neurobiol. Dis..

[26] Foley T.E., Fleshner M. (2008). Neuroplasticity of Dopamine Circuits after Exercise: Implications for Central Fatigue. NeuroMol. Med..

[27] Balthazar C.H., Leite L.H.R., Ribeiro R.M.M., Soares D.D., Coimbra C.C. (2010). Effects of Blockade of Central Dopamine D1 and D2 Receptors on Thermoregulation, Metabolic Rate and Running Performance. Pharmacol. Rep..

